# Nicotine-induced conditioned place preference involves dynamic changes in DNA methylation and hydroxymethylation in the zebrafish brain

**DOI:** 10.3389/fcell.2026.1841063

**Published:** 2026-08-20

**Authors:** J. Ortiz, L. Rocco, R. Bernabeu, M. P. Faillace

**Affiliations:** Department of Physiology, Institute of Physiology and Biophysics (IFIBIO, UBA-CONICET), School of Medicine, University of Buenos Aires (UBA), Buenos Aires, Argentina

**Keywords:** 5hmC mark, DNA demethylation, DNA methylation, nicotine reward, place preference, zebrafish

## Abstract

Modifications of the DNA methylation/demethylation cycle are fundamental for synaptic remodelling in memory-related processes. Cytosine methylation and demethylation marks epigenetically regulate gene expression and play key roles in addiction-like behaviours. DNA methyltransferases (DNMTs) catalyse methylation of DNA-cytosines (5mC), while ten-eleven translocation (TET) enzymes catalyse DNA demethylation to 5-hydroxymethylcytosine (5hmC) and further cytosine oxidations. 5hmC is stable and abundant in neurons and regulates gene expression. We examined whether conditioned place preference (CPP) to nicotine regulates 5mC and 5hmC levels in the zebrafish brain. Levels of TET1 mRNA were upregulated while levels of TET2, GADD45, DNMT, and MeCP2 mRNAs were downregulated in the zebrafish brain trained in the nicotine-CPP task. Levels of the *α6* and *α7* subunits of the nAChR and NMDAR1 mRNAs were also upregulated shortly after the nicotine-CPP test. Specific nuclei of the reward circuit, particularly the habenula (Hb) and raphe nucleus, and the tectum opticum (TeO) showed increases in the 5hmC mark in neuronal subpopulations induced by nicotine-CPP. Nicotine-CPP also increased, to a lesser extent, the number of cell nuclei containing high levels of 5mC in the Hb and TeO. Other reward structures, the posterior tubercular and interpeduncular nuclei, showed decreased numbers of cell nuclei containing high levels of 5mC with no changes in the number of cell nuclei showing high levels of 5hmC. Nicotine-CPP increased 5hmC levels and decreased 5mC levels in the whole brain of zebrafish. In this report, we demonstrate that the establishment of nicotine preference in the zebrafish brain is associated with changes in DNA methylation marks in neurons of the reward circuit, including the upregulation and downregulation of the 5mC mark, along with a net increase in the 5hmC mark. The specific changes in the methylation mark profile depend on the reward structure examined.

## 1 Introduction

Nicotine addiction is a persistent challenge despite concerted efforts to combat it. While there have been significant strides in tobacco control policies, public health campaigns, and smoking cessation programs, the addictive nature of nicotine continues to contribute to a substantial number of deaths worldwide each year (World Health Organization, 2021). In addition, smoking tobacco cigarettes could serve as a gateway drug, priming the use of more dangerous illicit substances (Levine et al., 2011). Smoking rates continue to decline worldwide, but nicotine vaporizers, especially among those who have never smoked, have replaced tobacco cigarettes. Nicotine vaping shows a higher prevalence of depression and anxiety than smoking (Kim and Picciotto, 2023). Therefore, nicotine addiction remains a health problem worldwide.

There is an interaction between drug-induced neuronal plasticity and epigenetic mechanisms, which contribute to the lasting behavioural changes, associated with substance use disorders (Bali and Kenny, 2019). Epigenetic mechanisms, such as DNA methylation and histone modifications, play a crucial role in regulating gene expression in response to drug consumption. Studies have demonstrated that addictive substances can induce epigenetic modifications in the structures of the brain reward circuit, leading to changes in the expression of genes involved in addiction-related behaviours (Koijam et al., 2024).

A predominant epigenetic mechanism in DNA is the methylation of cytosine at carbon 5. DNA methyltransferases (DNMTs) catalyse the methylation of cytosines in CpG sequences to 5-methylcytosine (5 mC) using S-adenosyl-L-methionine as the methyl donor (Rustad et al., 2019). A large family of proteins can bind methylated DNA. These methylated DNA-binding proteins are responsible for repressing gene transcription. A balanced activity of DNMTs -DNMT1, DNMT3a, and DNMT3b- and the ten-eleven translocation (TET) enzymes, a family of dioxygenases (TET1, TET2, and TET3), regulate dynamic changes in the DNA methylation/demethylation cycle (Joshi et al., 2022). Thus, TET proteins catalyse 5 mC oxidation to 5-hydroxymethylcytosine (5hmC), and successive demethylation steps to convert methylcytosines into 5-formyl (5fC) and 5-carboxylcytosines (5caC) (Guo et al., 2011). DNA modifications such as 5mC and 5hmC are stable, environmental sensitive, and highly enriched in the brain. It has been suggested that 5hmC is an epigenetic modification with an instrumental role as a long-lasting chromatin mark that regulates gene expression, rather than merely serving as an intermediate metabolite in the DNA demethylation cycle (Hahn et al., 2014; Chen et al., 2014). This makes 5hmC a potential contributor to epigenetically mediated responses to environmental factors. Furthermore, methyl CpG binding protein 2 (MeCP2), binds to 5mC but also to 5hmC. While the binding of MeCP2 to 5mC induces gene repression, its binding to 5hmC, which is enriched in active genes, facilitates gene transcription by organizing dynamic chromatin domains (Mellén et al., 2012).

Humans with lifetime nicotine exposure show blood cells with overall DNA hypomethylation, suggesting a more relaxed chromatin state and increased gene expression (Markunas et al., 2021; Liu et al., 2018). Following repeated administration of cocaine to mice which enhanced behavioural responses, TET1, but not TET2 and TET3, was downregulated in the nucleus accumbens, a key reward structure in the brain (Feng et al., 2015).

In addition, the gene coding for GADD45 (Growth arrest and DNA-damage-inducible), another enzyme involved in DNA demethylation, is induced by dopamine in the striatum and is necessary for cocaine reward memory (Zipperly et al., 2021). Nicotine exposure induces DNA demethylation in the brain, which was partially analysed in different species including zebrafish (Pisera-Fuster et al., 2021; Satta et al., 2008). Chronic administration of nicotine decreases DNMT1 expression in the frontal cortex and hippocampus of mice (Maloku et al., 2011; Satta et al., 2008), which was also observed in reward circuit nuclei of the zebrafish brain (Pisera-Fuster et al., 2021; 2020).

DNA demethylation catalysed by TET enzymes and 5hmC are actively involved in learning, memory, and drug-seeking behaviours, indicating that memory and addiction share overlapping molecular, cellular, and brain circuit mechanisms (Greer et al., 2021; Alaghband et al., 2016). Like humans, zebrafish exhibit behaviours related to drug reward, including the development of tolerance, relapse, and withdrawal syndrome (Klee et al., 2012; Stewart et al., 2011). The brain reward areas of zebrafish are homologous to mammalian brain regions with similar functions (Panula et al., 2010; Ninkovic and Bally-Cuif, 2006; Rink and Wullimann, 2001). Zebrafish possess a complete forebrain dopaminergic circuit similar to the mesolimbic system involved in drug addiction in mammals (Rink and Wullimann, 2002). The number of studies using zebrafish to assess drug reward has increased in recent years, demonstrating that they constitute a valuable model for evaluating the rewarding properties of substances involved in substance use disorders. Zebrafish have been used to assess the rewarding properties of nicotine using conditioned place preference (CPP) (Rocco et al., 2022; Pisera-Fuster et al., 2021; 2020; 2019; Faillace et al., 2018; Ponzoni et al., 2014; Kedikian et al., 2013; Kily et al., 2008). In zebrafish, nicotine not only induces a robust CPP, but also proves to be resistant to novel stimuli, such as choice-disrupting factors (Faillace et al., 2018). The *α7*, *β2*, and *α4* subunits of the nicotinic acetylcholine receptor (nAChR) are the most abundantly expressed in the brains of mammals and fish. They are critical for cognitive functions, including reward, whereas the α6 subunits are more specifically involved in nicotine behavioural effects and nicotine and cocaine-associated reward (Sanjakdar et al., 2015). Here we showed that nicotine-CPP altered the transcriptional expression of alpha subunits, suggesting that nicotine preference in zebrafish is at least partially mediated by upregulation of *α6* and *α7*-nAChR and NMDAR1 subunits. Only a few studies carried out in zebrafish have described epigenetic regulation in association with nicotine (Suresh et al., 2021; García-González et al., 2020; Pisera-Fuster et al., 2020; 2019; Kedikian et al., 2013; Petzold et al., 2009; Kily et al., 2008). Although there is abundant literature on DNA methylation in brain structures induced by addictive substances, relatively little research focuses on nicotine, and even less has been conducted in zebrafish (Pisera-Fuster et al., 2021; 2020). Most studies measured 5 mC under different conditions, and only one study evaluated 5hmC in relation to nicotine in the lungs of smokers (Caliri et al., 2020).

The use of a nicotine-induced CPP task allowed us to show that the rewarding effects of nicotine induce modifications of DNA methylation (5 mC) in the reward structures of the zebrafish brain, as we have evidenced in previous publications (Pisera-Fuster et al., 2021). In this study, we focused on the first enzymatic step of the DNA demethylation pathway that converts 5mC to 5hmC. We examined its occurrence in the nuclei of the reward circuit of zebrafish trained in the nicotine-CPP task. The findings described in this report demonstrated that specific changes in the DNA methylation/demethylation marks occurred in neuronal nuclei of the reward structures of the zebrafish brain. These changes were detected shortly after an associative learning process induced by nicotine exposure and expand our understanding of this widely conserved epigenetic mechanism.

## 2 Methods

### 2.1 Animal care and maintenance

Adult zebrafish (*Danio rerio,* Singapore strain; six to 9 months old) were obtained from a local farmer (Buenos Aires, Argentina) (Rocco et al., 2022). Zebrafish were maintained according to standard methods described previously (Westerfield, 2007). Zebrafish were kept in the housing tank, which consisted of a 120 L aquarium under a constant light/dark cycle of 14/10 h at 26 °C–28 °C, with aquatic plants and stone substrate and fed twice daily with dry food and *Artemia sp*.

All fish were acclimated to the laboratory facility for at least 15 days. After this period, the zebrafish were transferred to experimental housing tanks (12 x 20 x 15 cm), with 6-8 animals per tank, in the behavioural room. The experimental housing tanks were placed near the CPP tanks in the same room.

The manipulation of zebrafish in this study was previously described in detail elsewhere (Faillace and Bernabeu, 2016)**.** All animal work was conducted after approval from the Animal Research Ethics Committee of the University of Buenos Aires. Care was taken to minimize stress and the number of animals used in this work, in accordance with the ARRIVE guidelines. Animals were euthanized using terminal anaesthesia with tricaine methanesulfonate (MS-222, Sigma-Aldrich).

### 2.2 Drugs

Nicotine (nicotine hydrogen tartrate salt, Santa Cruz) was dissolved in aquarium water to produce a 30 μM solution according to our previous studies (Rocco et al., 2022; Faillace et al., 2018; Kedikian et al., 2013). Drug concentrations were calculated based on the weight of the salt.

### 2.3 Behavioural studies

#### 2.3.1 Conditioned place preference (CPP) procedure

The CPP was carried out according to our previous studies (Faillace and Bernabeu, 2016; Kedikian et al., 2013). The CPP tank had visual cues that divided the experimental tank into two-halves: one-half light-brown and the other half white with six black dots on the bottom. The water level was kept at 12 cm from the bottom of the tank to minimize stress. Briefly, CPP consisted in three sessions: 1) Pretest. On the first day, each fish was tested for baseline place preference for 10 min. The preferred compartment was defined as the compartment in which the fish spent the most time during the pretest. 2) Conditioning. On the second day, each zebrafish in the nicotine-paired group was first restricted to the preferred compartment (light-brown side) for 20 min and then to the white compartment, where it was exposed to 30 μM nicotine for 20 min. Zebrafish in the saline-treated group (saline-CPP) were handled in the same way as zebrafish in the nicotine group, but without exposure to nicotine in the white compartment. The conditioning was carried out over three consecutive days. 3) Test. On the fifth day, CPP was tested for each zebrafish in a drug-free environment, similarly to the pretest. The percentage of time they remained on each side of the tank was recorded for 10 min (test session). The tank was randomly rotated in the behavioural room during conditioning to avoid associations with spatial cues from outside the tank. Changes in place preference were determined using a score [score % = (percentage of the time spent in the non-preferred side during test) – (percentage of the time spent in the non-preferred side during pretest)]. Behaviour was recorded and videos were analysed manually and using Noldus Ethovision XT7 software (Noldus Information Technology, Netherlands, http://www.noldus.com). Both analyses were compared to reduce analysis errors.

#### 2.3.2 Behavioural analysis

A camera positioned approximately 1.0 m above the CPP tank was used to record video. All behavioral data were recorded and analysed during the last 10 min, after 2 min habituation to the CPP tank, during the pretest and test sessions. The motor activity and anxiety of the zebrafish were measured by analysing the following parameters: total time spent, number of entries, distance swum, and average velocity in the nicotine-paired compartment. The resulting recordings were analysed by direct observation and with Noldus Ethovision XT7 software. We assessed “time spent and number of entries into the nicotine-associated compartment” because they are important parameters that complement the CPP findings. Distance travelled (swum) and average velocity in the nicotine-paired compartment provided additional information on the behavioural effect of nicotine exposure and can be used to determine the anxiety-like behaviour of the fish (Tran et al., 2016; Stewart et al., 2015; Cachat et al., 2011).

### 2.4 Molecular studies

#### 2.4.1 Reverse transcription (RT) and quantitative real-time PCR (qPCR) analysis

The reverse transcription–polymerase chain reaction (RT-PCR) procedure was performed as previously described (Pisera-Fuster et al., 2021; 2019). Briefly, zebrafish from CPP experiments were euthanized and their brains were homogenized for RNA extraction using RNAzol reagent (Genbiotech, Buenos Aires, Argentina). Before homogenization, the olfactory bulb, cerebellum, rhombencephalon and most of the TeO were removed. RT-PCR and qPCR were run for each experimental group using the primers shown in Table 1. Total RNA (200 ng/well) was reverse transcribed with the reverse transcriptase MMLV (Invitrogen, Carlsbad, CA) and then, amplified by endpoint PCR (45 cycles) and qPCR (40 cycles), using the CFX connect real-time PCR detection system (Bio-Rad, Hercules, CA). Primers were generated using Primer Blast software from the zebrafish genome reported in the Ensembl database (Table 1). PCR products were examined using 2% agarose gels. The elongation factor 1α (*ef1α*) was analysed as the reference gene, in parallel with each gene of interest, and its mRNA levels showed no significant variations between the experimental groups. The 2^−ΔΔCt^ method was applied to identify the most suitable reference (housekeeping) gene under our experimental conditions (Vandesompele et al., 2002). Three candidate genes—*ef1α*, *βactin*, and *rps18*—were evaluated. The corresponding ΔCt values and ΔCt ratios were 0.06/1.003, 2.535/1.11, and 0.375/1.02, respectively. Thus, we used *ef1α* as the housekeeping gene because it showed virtually no variability between the experimental groups. Quantitative PCR analysis was performed using the “Gene expression’s CT difference” (GED) method (Schefe et al., 2006), which considers individual amplification efficiency, as previously detailed (Pisera-Fuster et al., 2021; 2019; Kedikian et al., 2013). PCR efficiency and CT values were calculated and averaged from triplicated PCR wells. The calibrator (saline-CPP) and interest samples (nicotine-CPP) were run in the same PCR assay for each target and housekeeping genes. NoRT samples (in which the MMLV was omitted of the RT reaction) were also analysed in triplicated wells, along with the calibration and interest samples. Amplification efficiencies for all samples were between 0.98 and 1.02. Results were also calculated by the 2^−ΔΔCT^ method, which considers 100% reaction efficiencies (E = 1.0). The data showed no significant differences compared to the values obtained with the GED analysis. Five independent pools were analysed per experimental condition. Pools of three brains were used due to the limited amount of tissue obtained from each zebrafish brain in the RNA extractions. The brains in each pool were obtained from animals that showed robust positive scores in the nicotine CPP task. The brains of zebrafish that clearly showed negative scores for CPP, in the absence of nicotine, were also pooled together for the saline-CPP group. Therefore, we were able to assess whether changes in mRNA expression could be associated with behavioural outcomes and reduced inter-assay variability by measuring the set of genes in the same cDNA samples.

**TABLE 1:** Primer pairs for determining the relative expression levels of various mRNAs under the experimental conditions.

Gene	Sense	Antisense
*efl1α, eef1a1l1-201* ENSDARG00000020850	TAC-CCT-CCT-CTT-GGT-CGC-TTT	CAG-CAC-CAC-CGA-TTT-TCT-TCT-C
*rps18-204-206* ENSDARG00000100392	AGA-ACC-CTC-GCC-AGT-ACA-AAA	TTG-TCC-AGA-CCA-TTA-GCA-AGG-A
*βactin, actb1* *ENSDARG00000037746*	GGC-AAT-GAG-CGT-TTC-CGT-TG	TAC-CGC-AAG-ATT-CCA-TAC-CCA-G
*dnmt1-201* ENSDARG00000030756.8	GGA-GGC-AGT-GGC-AGA-AGT-AA	CCA-TGT-TCT-CAT-CAT-CCT-CAG-C
*dnmt3ab-201* ENSDARG00000015566.12	AGA-ATA-CGA-GGA-TGG-CCG-AG	TCA-TCC-TCC-AGG-ATA-CGA-CC
*gadd45aa* ENSDARG00000043581	AGA-ACG-ACA-TCA-ACA-TCC-TGC	ATG-TGG-ACG-CAT-GTG-GAA-C
*tet1-201* ENSDARG00000075230.6	GCT-CTG-CAA-ATA-TGG-CTC-GC	AAT-GAG-AAA-GAG-GCA-CCG-CA
*tet2* *ENSDARG00000076928*	CTA-AAA-CGG-ACA-ACA-CCC-AGC	GTG-GAA-GAT-GCC-CAG-AGT-CAA
*mecp2-201* *ENSDARG00000014218*	GAC-GTC-TAC-CTT-ATC-AAC-CCA-G	CGT-GAA-GTC-AAA-GTC-ATT-GGG
*nachr α7 cr7, chrna7a-* ENSDARG00000101702	CCG-ACA-TCA-CAG-GAT-ACA-TTG-C	AGA-ACC-TCT-CAT-TCC-GTC-TAC-C
*nachr α6, chrna6-201* ENSDARG00000055559.4	TGT-CTG-ACC-CTG-TTA-CTG-TGG	CAT-CAA-ACT-CTG-CTG-GTG-ACC
*nachr α4 cr11, chrna4b-201* ENSDARG00000070724.3	TGA-GAA-TGT-CAC-CTC-CAT-CAG-G	TGA-GTC-ACC-GCA-AAG-TCT-CC
*nachr β2; chrnb2b-202* ENSDARG00000017790.10	TGG-AGC-CCA-GAA-GAG-TTT-GAT-G	ATA-GGA-GAC-GAC-AGC-ATT-GGA-G
*nmdar1, grin1a-201* ENSDARG00000027828.10	TCC-TAC-ACC-GCT-GGC-TTC-TA	GGT-GGG-AAT-ATG-GTG-GCA-CT

Primer pairs selected using exonic sequences of the zebrafish genome recognize annotated gene splicing variants. The gene identification (ID) number from the Ensembl database is shown.

### 2.5 Immunohistochemistry

Two hours after the CPP test, zebrafish were euthanized with anaesthetic and their brains were removed and fixed for 24 h in a cold fixative solution (90% ethanol, 5% formalin, 5% glacial acetic acid). Coronal cryosections 30 μm thick were prepared. Antibodies directed against 5 mC (rabbit mAb D3S2Z, catalogue #28692; 1:1,000; Cell Signaling, MA, United States) and 5hmC (mouse mAb HMC3, catalogue #51660; 1:600, Cell Signaling) were used. Briefly, cryosections were kept in blocking solution for 2 h at room temperature (RT) (0.1 M PBS with 0.1% Triton (PBS-T) and 5% horse serum). Subsequently, brain sections were incubated overnight at 4 °C with the primary antibody diluted in blocking solution. Then, sections were incubated with a biotinylated secondary antibody, against rabbit and mouse, made in horse (Jackson ImmunoResearch Labs, West Grove, PA, United States) for 2 h at RT and with avidin-biotin-peroxidase complex (ABC reagent, Vectastain Elite ABC Kit Universal, Vector Labs, Burlingame, CA, United States). Antibody detection was performed by using 3,3′-diaminobenzidine (DAB) and a nickel solution and H_2_O_2_ (Vectastain Elite ABC kit). Slices were dehydrated and coverslipped with permanent mounting medium. Both antibodies have been validated using ELISA, dot blot, and methylated-DNA immunoprecipitation assays and are expected to detect 5mC and 5hmC in DNA with high specificity in all species, including zebrafish (Cell Signaling datasheets; Johnson and Eno, 2026). The antibody against 5 mC has been used in rodents, humans, pigs, cattle, *Xenopus*, and zebrafish and the antibody against 5 hm C has been used in cell cultures, humans, rodents, and zebrafish. We performed appropriate controls in brain sections, including omission of the primary antibody and omission of the secondary antibody in the presence of the chromogenic compound (nickel-DAB).

### 2.6 Immunofluorescence

Cryosections were kept in blocking solution (PBS-T and 5% normal goat serum) for 2 h at RT. Then, they were incubated overnight at 4 °C with primary antibodies directed against 5mC (1:1000, rabbit mAb D3S2Z) and 5hmC (1:600, mouse mAb HMC3) diluted in blocking solution. To assess 5hmC expression in neurons, an antibody directed against NeuN (1:500; rabbit polyclonal antibody, Abcam, Cambridge; United Kingdom) diluted in blocking solution was used along with the antibody against 5hmC. After thorough washing, brain cryosections were incubated for 2 h at RT with Cy3 and Cy5-conjugated secondary antibodies, against mouse and rabbit antibodies, respectively, produced in goat (6 μg/mL; Jackson ImmunoResearch Labs). Sections were washed, mounted, and coverslipped in mounting medium for fluorescence (Vectashield, Vector Labs). Negative controls have been performed omitting primary or secondary antibodies. Fluorescent signal was detected using a confocal microscope (Laser Spectral Confocal FV1000, Olympus; Japan) and the images were processed with Adobe Photoshop. Only general contrast enhancements and colour level adjustments were made; the images were not digitally modified in any other way.

### 2.7 Cell counting

For quantification of fluorescent and nickel-DAB-labelled cells, we measured four areas of interest (AOI) of 2,500 μm^2^ per structure. Appropriate areas were digitally imaged and the brains of four animals per treatment were analysed. Each AOI was selected randomly in the posterior tuberal nucleus (PTN), habenula (Hb), interpeduncular nucleus (IPN), dorsal and ventral nucleus of the ventral telencephalic area (Vd/Vv), raphe nucleus (RN) and TeO. In the TeO, which we use as a control brain structure outside the reward circuit, we quantified cells outside the periventricular gray zone (PGZ), a neurogenic niche, which contains a very high density of neurons and glia, as well as stem/progenitor cells. Therefore, the number of cells counted per AOI in the TeO was significantly lower than the number of cells in most of the nuclei of the reward circuit computed in this study. The RN in zebrafish also contains a lower number of cells per AOI than the number of cells in the other reward nuclei.

The number of nickel-DAB-stained cells was determined using a transmitted light microscope (Neurolucida, Zeiss, Germany) and the optical dissector principle (Pisera-Fuster et al., 2019; 2021; Yurt et al., 2018; Bernabeu and Longo, 2010; Coggeshall and Lekan, 1996; Gundersen et al., 1988) for comparison between control and nicotine-CPP groups. The optical dissector principle is a stereological method used to obtain unbiased estimates of the number of particles (such as neurons or markers) which avoids the common counting biases that come from cell size, shape, or section thickness.

Quantification was performed using Image-Pro Plus (Media Cybernetics Inc., United States). For each animal, immunopositive cells were counted on three sections, which contained the brain structure examined (different brain nuclei and structures are localized in different sections and we used three sections per structure). Counts were averaged in squares of 2,500 μm^2^ drawn randomly in the PTN, Hb, IPN, Vd/Vv, RN and TeO. To define a cell as positive for a specific marker in each AOI, we selected a method with stringent criteria that has been previously reported (Pascual et al., 2009; Walters et al., 2005; Miller and Marshall, 2005). According to the stringent criteria we chose for this study, 5mC- and 5hmC-positive cell nuclei (cell nuclei exhibiting higher levels of 5mC and 5hmC) were defined as those showing levels of OD (nickel-DAB staining) or fluorescence (fluorescent antibodies) greater than 50% of the maximum OD/fluorescence above the minimum OD/fluorescence located within a cell nucleus (threshold). The software detected the minimum and maximum values of OD or fluorescence within cell nuclei in the AOI. The OD/fluorescence value of 50% was taken as the midpoint of the interval between maximum and minimum OD/fluorescence among all cells within the AOI. This 50% value was added to the OD/fluorescence threshold to obtain the cutoff value. The software automatically calculated the 50% and cutoff values from all stained nuclei (at any OD or fluorescence level) in the AOI. The minimum optical density or fluorescence was clearly distinguished from the acellular background.

### 2.8 Dot blot analysis

After DNA extraction from brain tissue (DNAzol, Molecular Research Center Inc., United States), the DNA concentration was determined using a Nanodrop spectrophotometer (NanoDrop LITE, Thermo Scientific) and diluted to a concentration of 50 ng/μL. The DNA samples were denatured at 99 °C for 5 min, cooled on ice, and centrifuged. Next, we mixed 2 µL of DNA (50 ng/μL) from each sample with 1 µL of ethidium bromide (EthBr, 1:1,000 of a 10 μg/μL stock solution). We then spotted 2 µL of the DNA/EthBr solution onto a nitrocellulose or nylon membrane (Hybond ECL or N+, respectively, Amersham, MA). The membranes were air-dried for 15 min and the DNA was cross-linked by irradiation with 302 nm UV light for 5 min. Images of the EthBr-labelled DNA were taken to document the amount of DNA loaded at each spot. Next, we removed EthBr from the DNA spots by washing the membranes with 1 mL of a solution containing 20% ethanol: 80% dH_2_O for 20 min at RT with agitation. The membrane-bound DNA was blocked with 5% fat-free milk powder in PBS-T for 1 h at RT and incubated overnight at 4 °C with either 5mC or 5hmC primary antibodies (1:1,000 in blocking solution). The membrane-bound DNA was washed three times with PBS-T for 10 min and incubated with a universal biotinylated secondary antibody (1:100 in blocking solution) for 2 h at RT. Then, the ABC reagent (50 μL/5 mL; Vectastain Elite, Vector labs) was applied for 2 h at RT and DAB and nickel were used as chromogen. Developing time of nickel-DAB was followed over time and we selected an adequate time point (15–20 s for 5mC and 60 s for 5hmC) to avoid reaching dot saturation. Images of the stained spots were acquired using a G-box (Syngene) and GenSnap software. ODs were determined using Gel Pro Analyzer 4.0 software (Media Cybernetics), subtracting the background OD as a correction for dots of interest and then normalizing the OD to the loading control. To determine intensity relative changes (fold change), the mean OD of the control group was established as a reference value, and the OD of each sample of interest was compared to this reference value. We omitted the primary antibodies as negative controls and did not obtain a labelling distinct from that of the membrane background. For additional negative controls, we used a horseradish peroxidase (HRP)-conjugated secondary antibody against mouse (Jackson Labs) to detect the rabbit mAb D3S2Z for 5 mC detection. We also used a HRP-coupled secondary antibody against rabbit (Jackson Labs) to detect the mouse mAb HMC3 for 5hmC detection. When we incubated the membrane with nickel-DAB and H_2_O_2_, we did not observe any labelling distinct from the background of the membrane, regardless of the development time. When we used the correct combination of rabbit HRP-conjugated primary antibody against rabbit and mouse HRP-conjugated primary antibody against mouse, we obtained positive labelling in cell nuclei in the presence of the chromogenic solution.

### 2.9 Statistical analysis

CPP scores were analysed using Student’s t-test. Behavioural parameters such as total time spent, number of entries, total distance swum and average velocity in the nicotine-paired compartment of the CPP-tank were analysed by two-way ANOVA (pretest vs. test values, as repeated measures). The scientists involved in the analysis of RT-qPCR data, dot blot quantification, and immunohistochemical cell counts were blinded to all treatments. Normal distribution of data was analysed by D´Agostino and Pearson omnibus normality test. RT-qPCR data were analysed using Student’s t-test. Dot blot quantification was analysed using an unpaired Student’s t-test. Both qPCR and dot blot quantification were expressed with t, degrees of freedom (*df*) and p-value, confidence interval (CI) 95% to provide the interval where plausible population value could be contain and *R* ^2^ to assess the magnitude of the treatment. For cell number quantification in IHC studies labelled with nickel-DAB, statistical analyses were performed using one-way ANOVA. Comparisons of double-positive cell nuclei counts were performed using two-way ANOVA. ANOVA tests were followed by Bonferroni test *post hoc* comparisons. Data were presented as the mean ± SD and significance was set at p < 0.05. Alpha values are indicated in the legend of each figure. All data were plotted and analysed with GraphPad Prism 8.0.

## 3 Results

### 3.1 Behavioural characterization of nicotine-induced CPP in adult zebrafish

#### 3.1.1 Conditioning place preference

**FIGURE 1:**
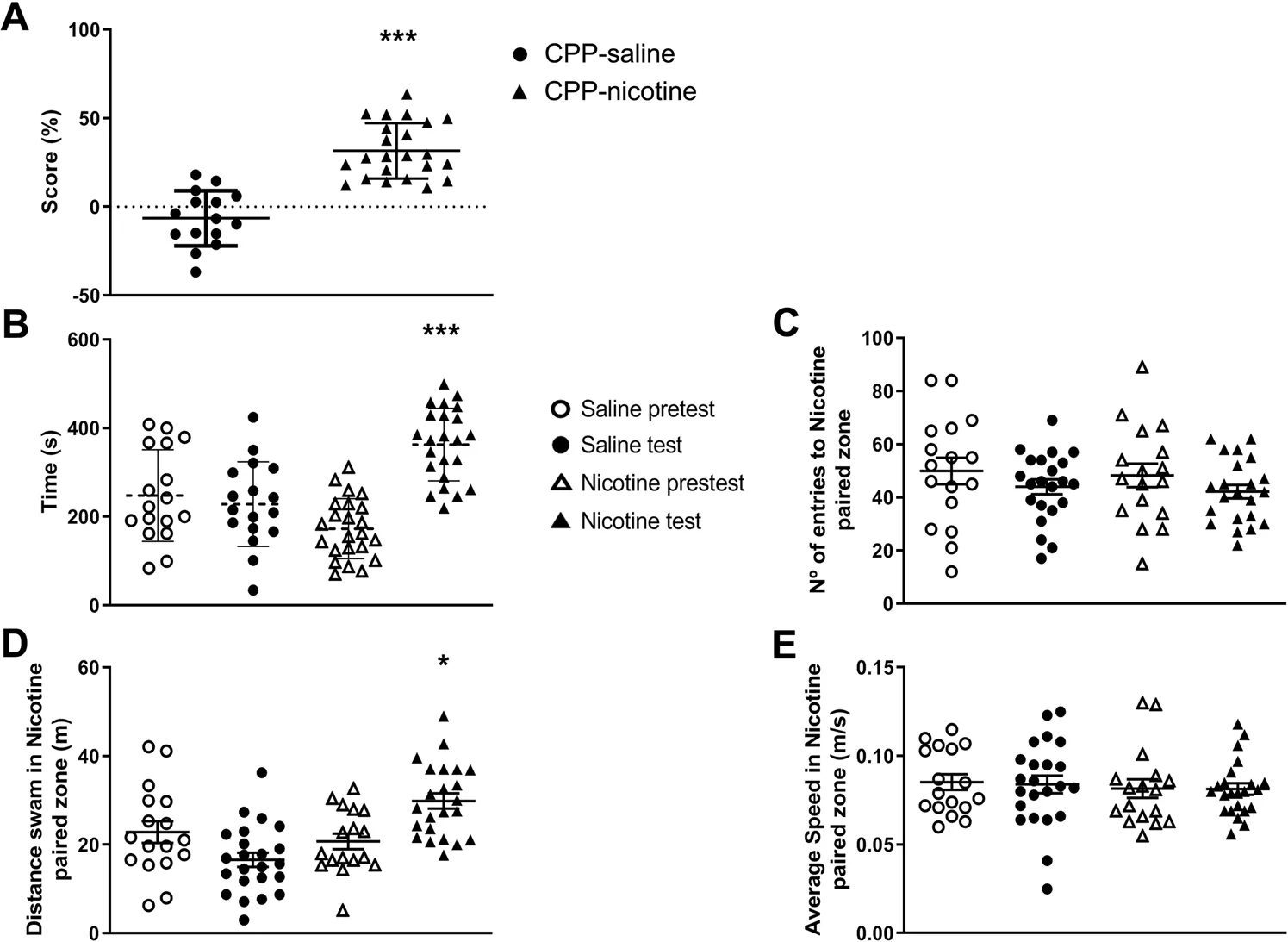
Behavioural analysis of zebrafish trained in a nicotine-induced CPP task. **(A)** Positive CPP scores were obtained by using 15 mg/L nicotine diluted in the non-preferred compartment of the CPP tank. CPP score was calculated as: % of time spent in the drug-paired side after drug exposure (test) minus % of time spent on the drug-paired side before drug exposure (pretest) over a 10-min period. **(B)** The graph shows the time spent in the white compartment (drug-paired zone) during pretest and test sessions for individual zebrafish from the two experimental groups. A series of behavioural parameters were also analysed during the CPP task: **(C)** Number of entries to the nicotine-paired zone, **(D)** distance swum in the nicotine-paired zone and **(E)** average speed in the nicotine-paired zone. Data are presented as mean ± SD (saline-CPP task; n = 17 zebrafish); 15 mg/L nicotine (nicotine-CPP task; n = 23 zebrafish) *p < 0.05; ***p < 0.001.

To evaluate whether zebrafish established an association between nicotine reward and the place where nicotine was administered, we trained the zebrafish in nicotine-CPP and saline-CPP tasks. Zebrafish were tested in the CPP task the day after conditioning in nicotine-free environments to evaluate changes in place preference induced by nicotine reward, and CPP scores were calculated as described in the Methods section. Figure 1A shows saline and nicotine-CPP scores. Student’s t-test revealed a significant increase in the CPP-score in fish exposed to nicotine compared to zebrafish exposed to saline solution in the CPP tank (t (36) = 7.352, p < 0.0001). Zebrafish in the nicotine-CPP group showed significantly positive scores indicating a robust nicotine-environment association.

#### 3.1.2 Behavioural analysis of zebrafish during the nicotine-CPP task

We performed additional behavioural analysis during the pretest and test session of the saline and nicotine-CPP tasks, in the absence of nicotine, to examine whether nicotine conditioning modified zebrafish´s preference associated behaviours, such as locomotor activity, time and number of entries to the drug-associated compartment during conditioning sessions. Figure 1B shows the total time spent by zebrafish in the nicotine-paired side during the CPP task. Two-way ANOVA with repeated measures revealed no significant effect of pretest/test (F_1,38_: 1.05; p = 0.31) but very significant effects of CPP (F_1,38_: 25.71; p < 0.0001) and pretest/test × CPP interaction (F_1,38_: 38.07; p < 0.0001). Zebrafish trained in the CPP task without nicotine showed no significant differences in the time spent in each compartment. Figure 1C shows no changes in the number of entries to the nicotine-paired or to the non-preferred compartment between experimental groups. Two-way ANOVA revealed no significant effects of pretest/test (F_1,37_: 2.26; p = 0.14), CPP (F_1,37_: 0.3; p = 0.59) and pretest/test × CPP interaction (F_1,37_: 0.0007; p = 0.98). The distance swum was significantly increased in zebrafish trained in the nicotine-CPP task, which was particularly observed in the nicotine-paired compartment, during test sessions (D). Two-way ANOVA revealed no significant effects of pretest/test (F_1,38_: 0.44; p = 0.51) but significant effects of CPP (F_1,38_: 13.10; p = 0.0009), and pretest/test × CPP interaction (F_1,38_: 24.76; p < 0.0001). The average speed of the zebrafish in the CPP tank was analysed before (pretest) and after conditioning (test; E). No significant changes were found between the experimental groups by two-way ANOVA (pretest/test (F_1,38_: 0.02; p = 0.89), CPP (F_1,38_: 1.03; p = 0.32), and pretest/test × CPP interaction (F_1,38_: 0.03; p = 0.87)).

### 3.2 Transcriptional regulation of genes encoding nAChR and NMDAR subunits and epigenetic enzymes involved in DNA methylation and demethylation in zebrafish subjected to nicotine-CPP

**FIGURE 2:**
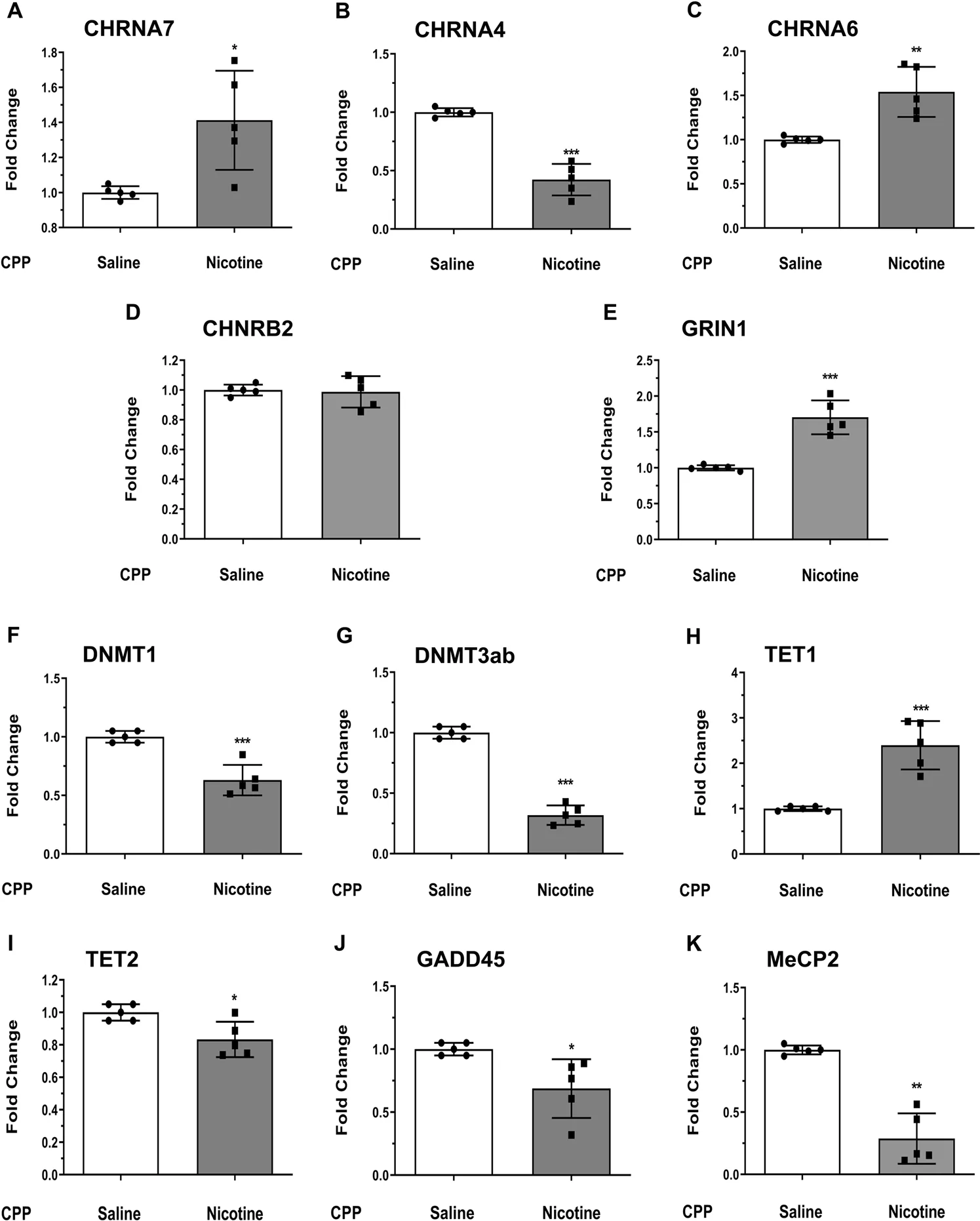
Transcriptional expression of nAChR and epigenetic enzymes involved in DNA methylation in zebrafish trained in the nicotine-CPP task. Relative mRNA levels were quantified by RT-qPCR in portions of the zebrafish brain containing the reward circuit structures (cerebellum, TeO, rhombencephalon, and olfactory bulb were removed). Zebrafish were euthanized 2 h after undergoing the saline or nicotine-CPP test. Graphs show the relative expression level (fold change) of nAChR-*α7* (CHRNA7, **(A)**, *α4* (CHRNA4, **(B)**, *α6* (CHRNA6, **(C)**, *β2* (CHRNB2, **(D)**, NMDAR1 (GRIN1, **(E)**, DNMT1 **(F)**, DNMT3AB **(G)**, TET1 **(H)**, TET2 **(I)**, GADD45 **(J)**, MeCP2 **(K)** mRNAs. Data are expressed as the mean ± SD (n = 5 pools of three brains each, per experimental condition). *p < 0.05, **p < 0.01 and ***p < 0.001, using unpaired t-test.

To examine whether nicotine-CPP induced changes in the transcriptional expression of genes that encompass a critical role in the establishment of nicotine reward, we determined the relative levels of nAChR subunits and the subunit 1 of the glutamatergic NMDA receptor (GRIN1) in part of the zebrafish brain containing the structures of the reward circuit. These changes were determined by RT-quantitative PCR two hours after the nicotine-CPP test. Figure 2 shows the relative expression of mRNA levels of *α*_*4*_ *, α*_*6,*_ *α*_*7*_ and *β2* subunits of the nAChR (CHRNA4, CHRNA6, CHRNA7 and CHRNB2, respectively) and of GRIN1. All receptor subunits, except CHRNB2, showed significant differences between the saline and nicotine-CPP groups (CHRNA4: t (8) = 9.23, p < 0.0001, CI: −0.7205 to −0.4324; *R* ^2^: 0.9141) (CHRNA7: t (8) = 3.237, p = 0.0119, CI: 0.1187 to 0.7065, *R* ^2^: 0.5671) (CHRNA6: t (8) = 4.24, p = 0.0028, CI: 0.247 to 0.8361, *R* ^2^: 0.6920) (CHRNB2: t (8) = 0.2418, p = 0.815, CI: −0.1269 to 0.1028; *R* ^2^: 0.007254); and (GRIN1 (t (8) = 6.592, p = 0.0002, CI: 0.4582 to 0.9513, *R* ^2^: 0.8445). The relative expression levels of the CHRNA7 and CHRNA6 mRNA showed a significant increase (A, C), whereas the transcriptional expression of the CHRNA4 decreased and the CHRNB2 showed no change in the nicotine-conditioned group (B, D). The relative expression levels of GRIN1 mRNA were also upregulated by nicotine-CPP (E).

To investigate whether nicotine-CPP modified the transcriptional expression of epigenetic enzymes involved in the regulation of the different steps of the DNA methylation and demethylation cycle, we analysed the relative expression levels of DNMT, TET, and GADD45 mRNAs in part of the zebrafish brain containing the reward nuclei. We also analysed the transcriptional expression of MeCP2, an important transcriptional regulator that binds to 5mC and 5hmC with high affinity. We found significant effects of nicotine-CPP on the relative expression levels of DNMT1 (t (8) = 5.946, p = 0.0003, CI: −0.5129 to −0.2263, *R* ^2^: 0.8155); DNMT3ab (t (8) = 16.08, p < 0.0001, CI: −0.7791 to −0.5836, *R* ^2^: 0.97); TET1 (t (8) = 5.832, p = 0.0004, CI: 0.8455 to 1.952, *R* ^2^: 0.8096); TET2 (t (8) = 3.094, p = 0.0148, CI: −0.2904 to −0.04237, *R* ^2^: 0.5448); GADD45 (t (8) = 2.927, p = 0.0191, CI: −0.5578 to −0.06621, *R* ^2^: 0.5172; MeCP2 (t (8) = 7.743, p = 0.0012, CI: −0.9243 to −0.5001; *R* ^2^: 0.8823). The expression levels of DNMT1 and DNMT3ab mRNA were decreased in the brain of zebrafish with positive scores in the nicotine-CPP task (F, G). The expression level of TET1 mRNA was significantly increased (H) in the nicotine-CPP group, while expression levels of TET2, GADD45 and MeCP2 mRNAs were significantly decreased in the brain of zebrafish trained in the nicotine-CPP task (I, J, K).

### 3.3 Changes in the level of DNA methylation and hydroxymethylation in the whole brain of zebrafish trained in a CPP task

**FIGURE 3:**
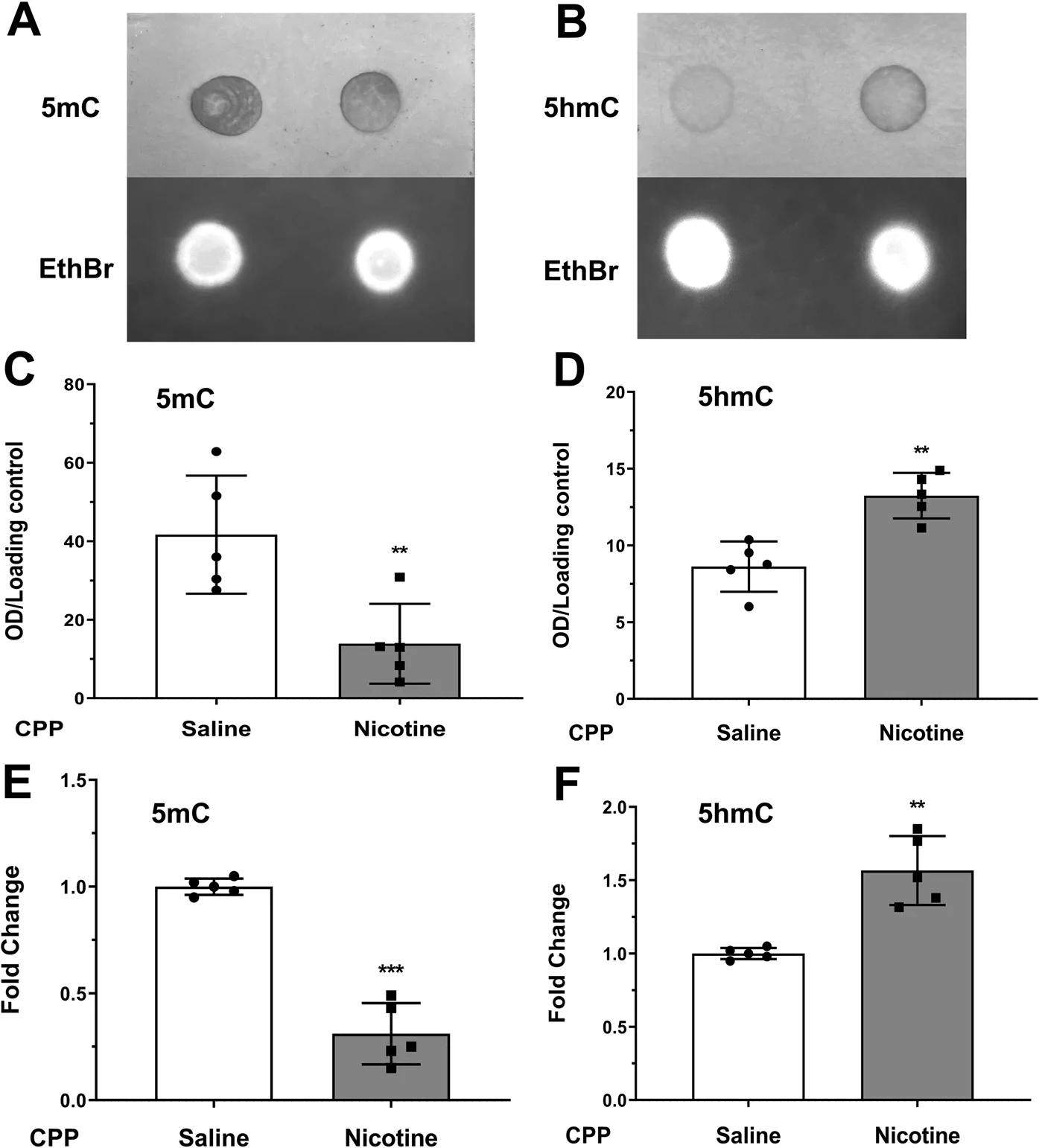
Global levels of cytosine methylation and hydroxymethylation were detected in the DNA of the brain of zebrafish trained in the nicotine-CPP task. Dot blots of DNA, purified from whole brain homogenates, were performed on nitrocellulose membranes. **(A)** DNA dot blot images obtained with a specific antibody against 5 mC (upper dots) and loading control images stained with ethidium bromide (lower dots), which were taken before performing the immunostaining. **(B)** DNA dot blot images obtained with a specific antibody against 5hmC (upper dots) and loading control images (lower dots) (see Methods for details). The changes in optical density (OD) and relative OD between experimental groups (fold change) are shown for 5mC (**(C,E)**, respectively) and for 5hmC (**(D,F)**, respectively). Data are presented as the mean ± SD, from independent experiments (n = 5 zebrafish brains per experimental condition). **p < 0.01 and ***p < 0.0001, using unpaired t-test for OD levels and fold change.

We first aimed to assess whether nicotine-CPP induced global changes in 5 mC and 5hmC, which are stable and abundant epigenetic marks regulating gene expression, in the brain of zebrafish shortly after conditioning. Purified DNA from zebrafish whole brain homogenates was used in dot-blot assays using specific antibodies against 5mC and 5hmC (Figures 3A,B, respectively). Although dot blot is a semi-quantitative technique, we have consistently detected that the levels of 5 mC decreased and the levels of 5hmC increased in the brains of zebrafish with positive scores in the nicotine-CPP task (Figures 3C–F). OD levels and relative changes in OD between treatments (fold change) of 5 mC decreased significantly in the brains of zebrafish trained in the nicotine-CPP task (t (8) = 3.424, p = 0.009, CI: −46.52 to −9.078, *R* ^2^: 0.5944 and t (8) = 10.35, p = 0.0002, CI: −0.8649 to −0.5125, *R* ^2^: 0.9592, respectively) (C, E). OD levels and relative OD levels of 5hmC significantly increased in the nicotine-CPP group compared to the saline-CPP group (t (8) = 4.68, p = 0.0016, CI: 2.344 to 6.899, *R* ^2^: 0.7325 and t (8) = 5.322, p = 0.0052, respectively) (D, F).

### 3.4 Immunostaining images of cells positive for 5mC and 5hmC in nuclei of the reward circuit of zebrafish trained in nicotine and saline-CPP tasks

To determine whether nicotine-CPP induced changes in the expression pattern of 5mC and 5hmC in cell nuclei of specific reward circuit structures and TeO in the zebrafish brain, we assessed these stable DNA epigenetic marks by IHC and nickel-DAB staining. The Vd/Vv, PTN, Hb, IPN, and RN were selected because they encompass the dopaminergic mesolimbic reward circuit in the zebrafish brain. TeO is a brain structure, homologous to the mammalian superior colliculus, with associative polymodal sensory function (Reichenthal et al., 2018).

**FIGURE 4:**
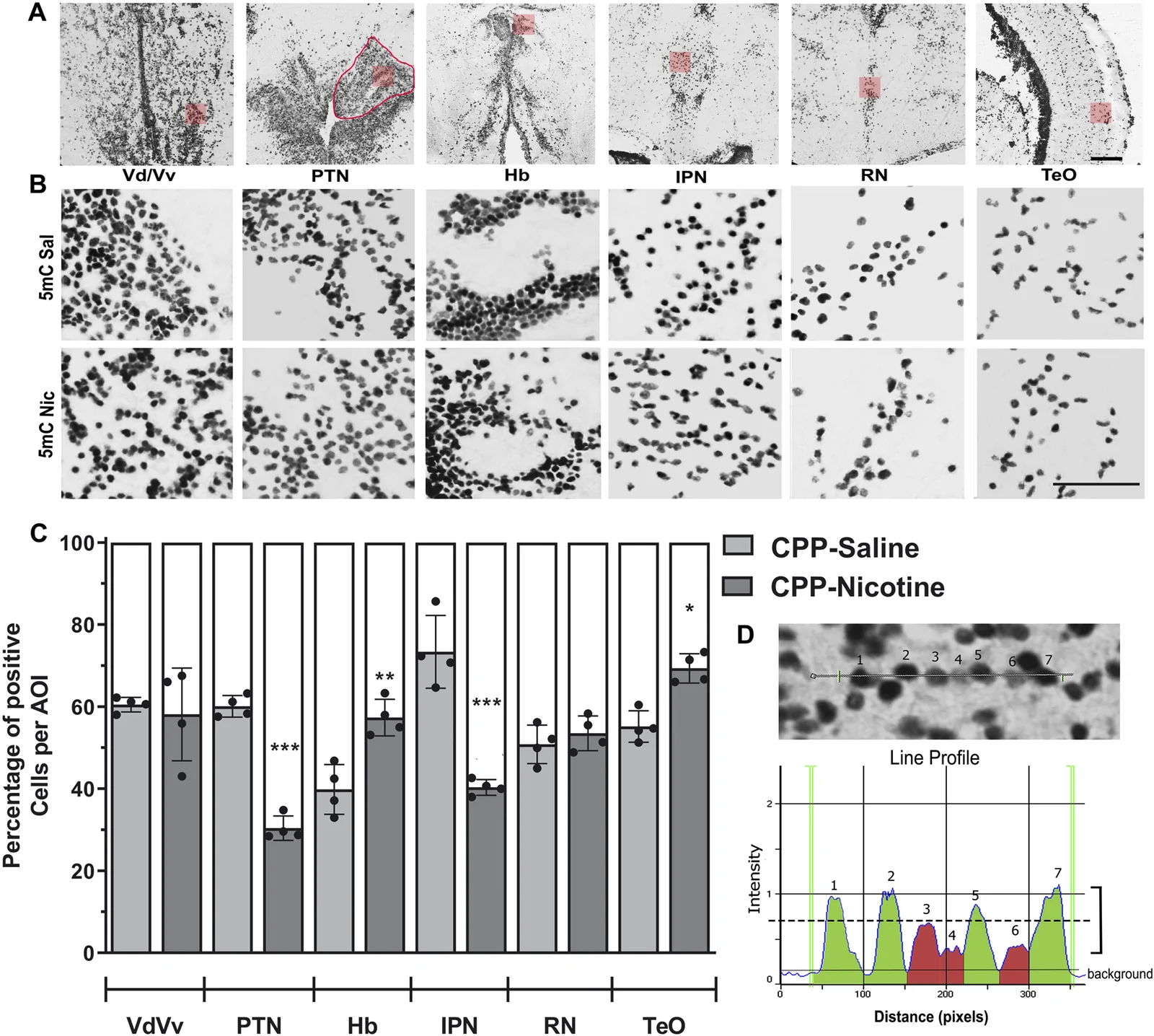
Quantification of 5mC-positive cells in areas of the reward circuit and other brain nuclei of zebrafish trained in the nicotine-CPP task. **(A)** Photographs of zebrafish brain sections highlighting the areas of interest (AOIs) where cells positive for epigenetic marks were quantified. Each image (40x magnification) shows the nucleus or region examined—specifically the Vd/Vv: dorsal and ventral nuclei of the ventral telencephalic area, PTN: posterior tuberal nucleus, Hb: habenula, IPN: interpeduncular nucleus, RN: raphe nucleus, and TeO: tectum opticum. Brain sections were analysed using *Neuroanatomy of the Zebrafish Brain*: A Topological Atlas (Wullimann et al., 1996). This atlas provided the detailed neuroanatomical parameters essential for the accurate identification and localization of each brain region. **(B)** Photomicrographs revealing 5mC-positive cells detected by immunohistochemistry and nickel-DAB in the Vd/Vv, PTN, Hb, IPN, RN, and TeO. **(C)** Bar graphs indicate the percentage of 5mC-positive cells relative to the total number of cells in each structure. **(D)** higher-magnification insets and the profile of several nuclei detected as positive (above 50 % cut off values) by the counting algorithm. Data are presented as the mean ± SD (n= 4 zebrafish per experimental group). *p < 0.05, **p < 0.01 and ***p < 0.001. One-way ANOVA followed by Bonferroni post hoc test. Scale bar: 50 μm.

Figure 4A depicts representative images of 5 mC-positive cell nuclei in the Vd/Vv, PTN, Hb, IPN, RN and TeO of the zebrafish brain. Since 5mC and 5hmC are epigenetic marks present in virtually all cells of different tissues, and 5hmC is especially abundant in the nervous system, we would not have been able to observe any effect on these marks without applying stringent criteria (described in Methods). These criteria were applied to select cell subpopulations that had significantly increased their 5mC and 5hmC levels due to nicotine-induced CPP. When quantifying all cell nuclei positive for 5mC or 5hmC, at any level, we observed no changes in the number of such cell nuclei between treatments.

**FIGURE 5:**
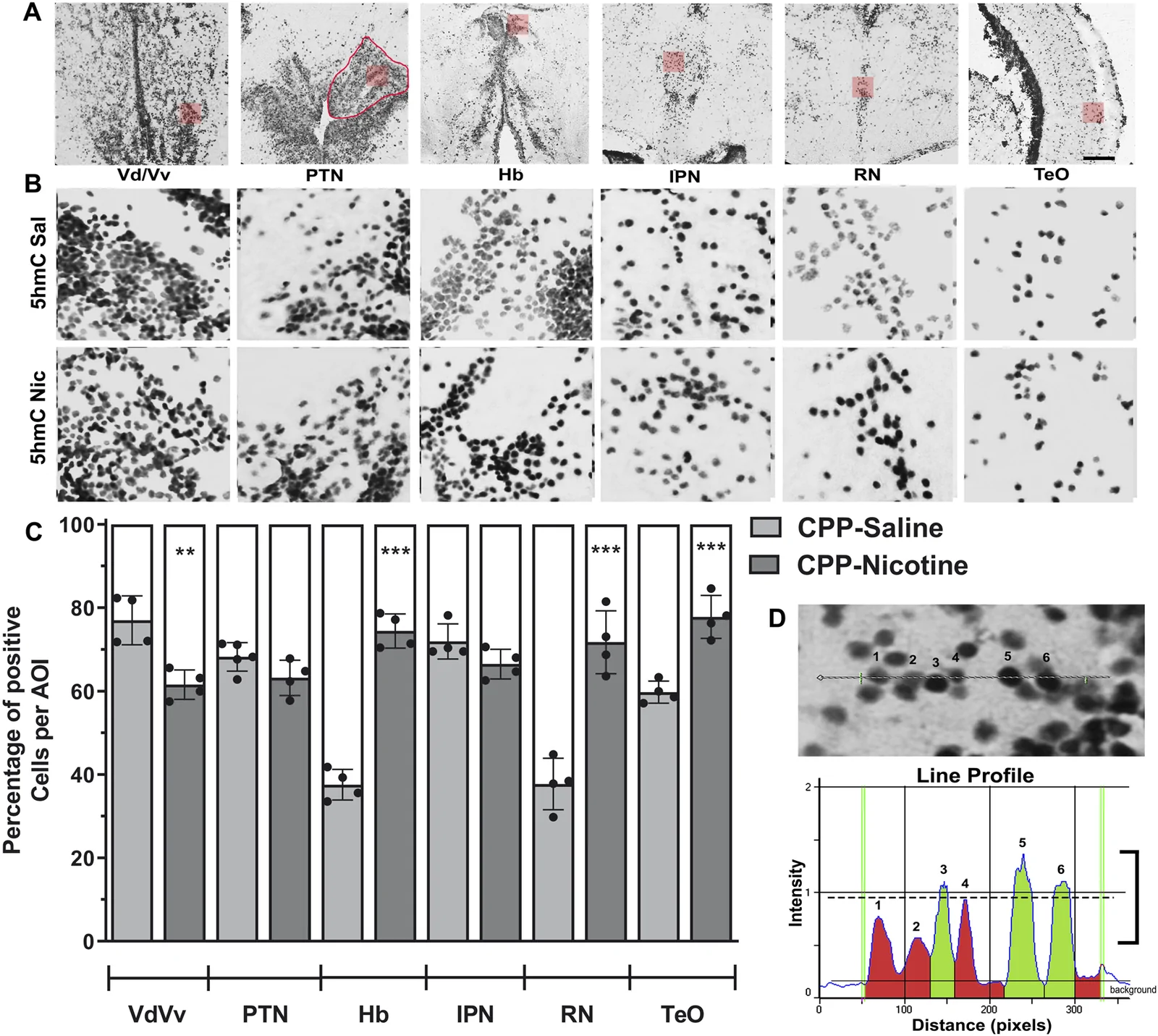
Quantification of 5hmC-positive cells in areas of the reward circuit and other brain nuclei of zebrafish trained in the nicotine-CPP task. **(A)** Photographs of zebrafish brain sections highlighting the areas of interest (AOIs) where cells positive for epigenetic marks were quantified. Each image (40x magnification) shows the nucleus or region examined—specifically the Vd/Vv: dorsal and ventral nuclei of the ventral telencephalic area, PTN: posterior tuberal nucleus, Hb: habenula, IPN: interpeduncular nucleus, RN: raphe nucleus, and TeO: tectum opticum. Brain sections were analysed using Neuroanatomy of the Zebrafish Brain: A Topological Atlas (Wullimann et al., 1996). This atlas provided the detailed neuroanatomical parameters essential for the accurate identification and localization of each brain region. **(B)** Photomicrographs revealing 5hmC-positive cells detected by immunohistochemistry and nickel-DAB in the Vd/Vv, PTN, Hb, IPN, RN, and TeO. **(C)** Bar graphs indicate the percentage of 5hmC-positive cells relative to the total number of cells in each structure analysed. **(D)** higher-magnification insets and the profile of several nuclei detected as positive (above 50 % cut off values) by the counting algorithm. Data are presented as the mean ± SD (n = 4 zebrafish per experimental group). **p < 0.01 and ***p < 0.001. One-way ANOVA followed by Bonferroni post hoc test. Scale bar: 50 μm.

The number of cell nuclei containing high levels of 5 mC changed significantly after nicotine conditioning in some of the brain nuclei examined. Data obtained from four brains (n = 4, zebrafish per experimental condition) were analysed by ANOVA: F_11,36_: 20.68; p < 0.0001, Figure 4B). Nicotine-CPP induced a significant increase in the number of cell nuclei expressing high levels of 5 mC by 19% in Hb and 15% in TeO, while decreasing the number of such cell nuclei in the PTN and IPN by 30% and 35%, respectively. No significant differences were found in this parameter in the Vd/Vv and RN between treatments. Figure 5A depicts representative images of 5hmC-positive cell nuclei in the Vd/Vv, PTN, Hb, IPN, RN, and TeO in the zebrafish brain. Figure 5B shows that the number of cells expressing high levels of 5 hmC changed significantly after nicotine-CPP test in the Vd/Vv, Hb, RN and TeO (ANOVA: F_11,37_: 33.73; p < 0.0001; n = 4 zebrafish per experimental condition). Nicotine-CPP caused a significant increase in the number of cell nuclei showing high levels of 5hmC by 36% in Hb, 33% in RN and 19% in TeO, while decreasing the number of such cell nuclei in Vd/Vv by 15%. No significant differences were found in this parameter in the PTN and IPN between zebrafish trained in saline or nicotine-CPP tasks.

**FIGURE 6:**
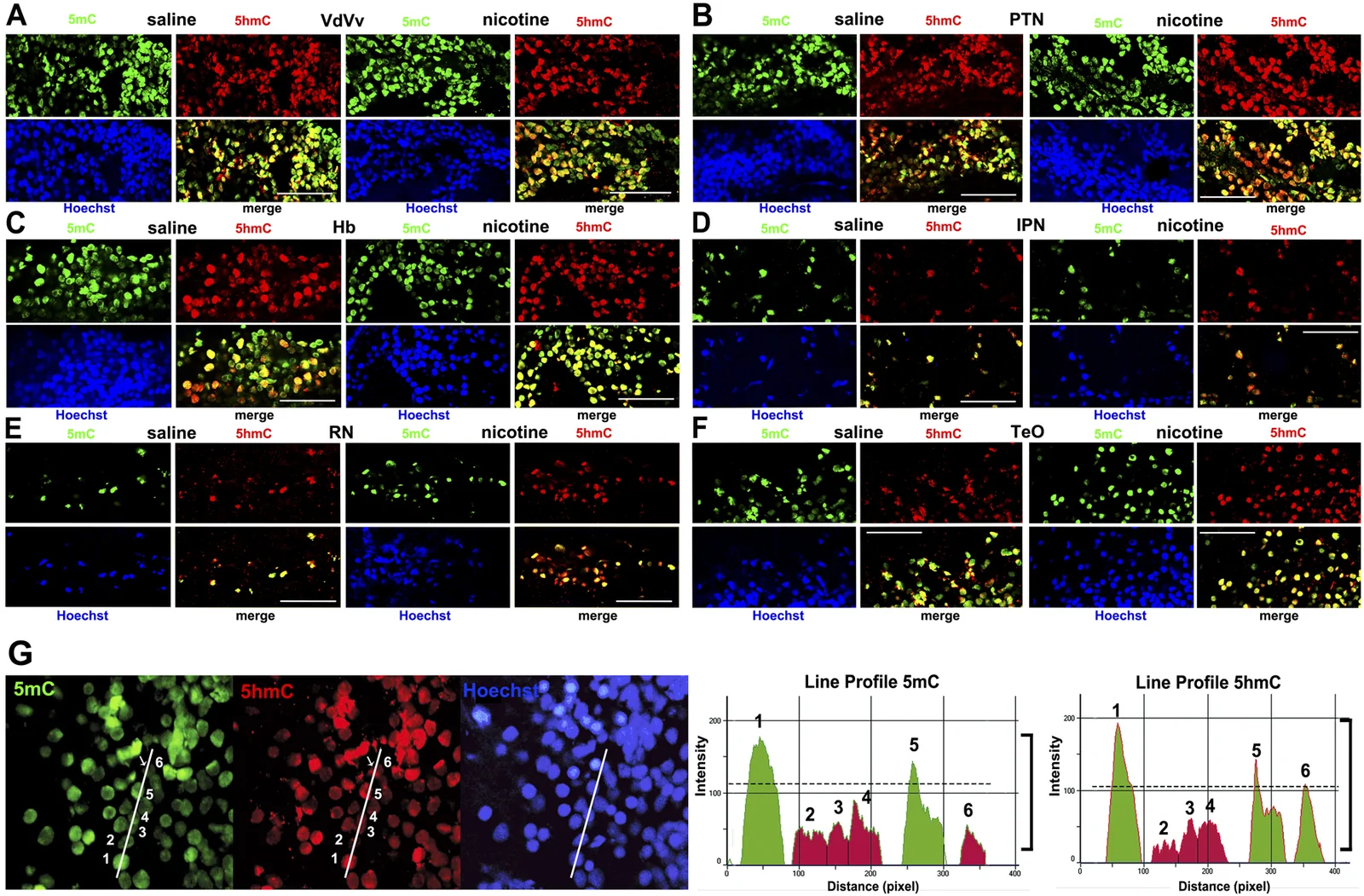
Fluorescent confocal images of 5mC and 5hmC in areas of the reward circuit and other brain nuclei of zebrafish trained in the nicotine-CPP task. Confocal images revealing 5mC (left column) and 5hmC (right column)-positive cells in the dorsal and ventral nuclei of the ventral telencephalic area (Vd/Vv) **(A)**, posterior tuberal nucleus (PTN) **(B)**, habenula (Hb) **(C)**, interpeduncular nucleus (IPN) **(D)**, raphe nucleus (RN) **(E)** and tectum opticum (TeO) **(F)**. The green and red labelling shows cell nuclei positive for 5 mC and cell nuclei positive for 5hmC, respectively. Hoechst-stained nuclei and double-positive nuclei for 5mC and 5hmC are shown in blue and yellow, respectively (n = 4 zebrafish per experimental group). **(G)** Higher magnification images and profile of nuclei detected as positive (pink, above the 50% cut off value) and negative (green) by the counting algorithm. Brackets indicate the range of cell nuclei positive for 5 mC and 5hmC, from minimum to maximum specific staining; the dotted line indicates the cut off value. The arrow indicates a 5hmC-positive nucleus, which is negative for 5mC. These nuclei were not quantified as positive cell nuclei in Figure 3. Scale bars: 50 µm.

**FIGURE 7:**
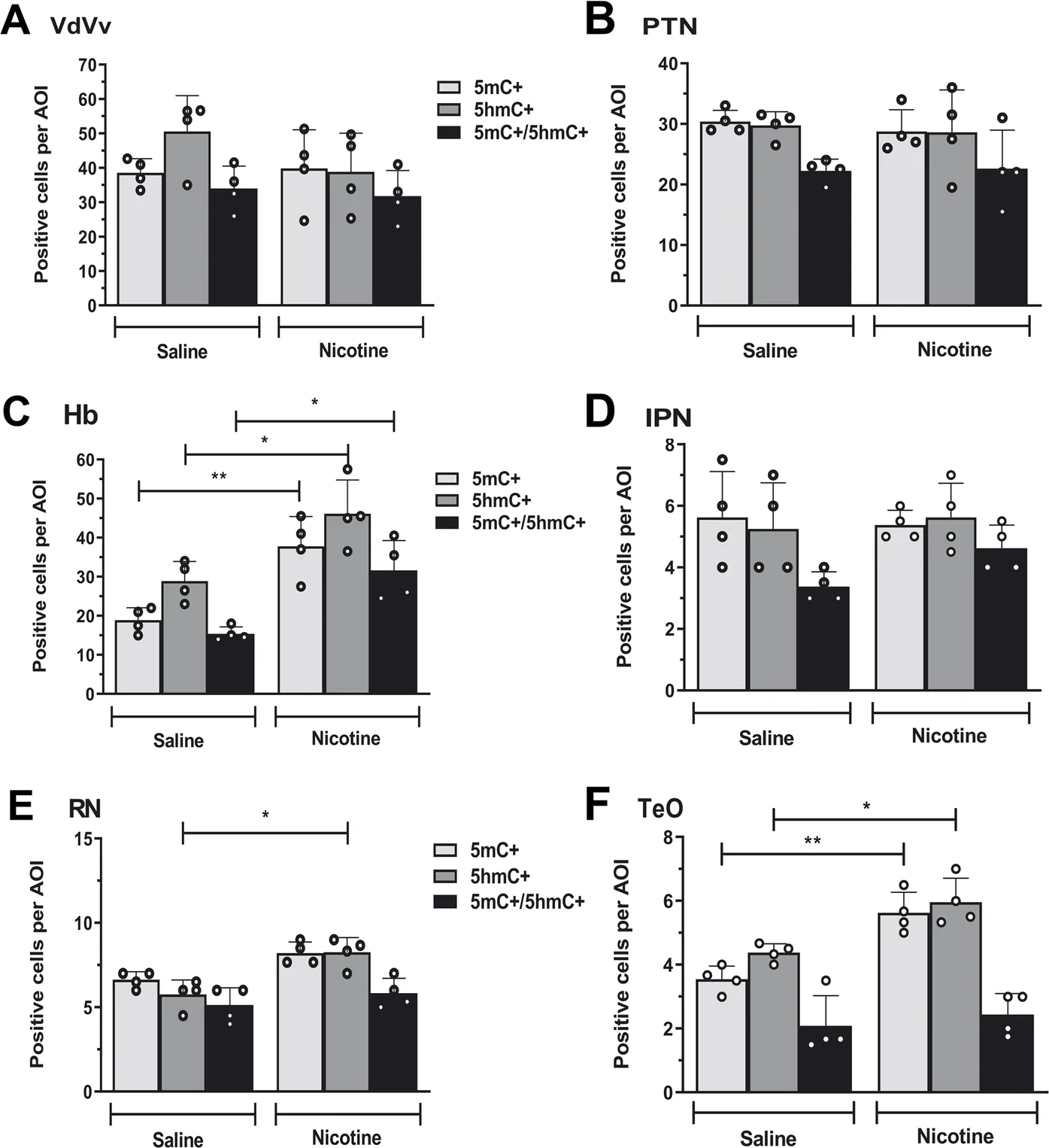
Quantification of 5mC and 5hmC positive cells in fluorescent confocal images of brain areas of zebrafish trained in the nicotine-CPP task. Bar graphs indicate the percentage of cells expressing 5mC, 5hmC, and both epigenetic marks (double-positive nuclei) in areas of interest (AOI) in the: **(A)** dorsal and ventral nuclei of the ventral telencephalic area (Vd/Vv), **(B)** posterior tuberal nucleus (PTN), **(C)** habenula (Hb), **(D)** interpeduncular nucleus (IPN), **(E)** raphe nucleus (RN), and **(F)** tectum opticum (TeO). *p < 0.01 and ***p < 0.001. Data are presented as the mean ± SD (n = 4 zebrafish per experimental group). Two-way ANOVA followed by Bonferroni *post hoc* test.

Next, we evaluated the number of cell nuclei that showed 5mC and 5hmC marks in the reward circuit structures using fluorescent immunostaining (Figure 6). Confocal microscopy images revealed cell nuclei doubly positive for both epigenetic marks. In virtually all structures analysed, a large proportion of 5mC-positive cell nuclei showed immunofluorescence for 5hmC. The number of cell nuclei positive for 5mC and 5hmC and the number of cell nuclei positive for both epigenetic marks were estimated and shown in Figure 7. The number of cell nuclei expressing higher levels (using the stringent criteria defined in Methods) of 5mC and 5hmC marks exhibited differences among zebrafish trained in the saline and nicotine-CPP tasks in three of the six brain nuclei analysed. Nicotine-CPP caused increases in the number of cell nuclei expressing high levels of 5mC, 5hmC and both marks in the Hb (C). Nicotine-CPP induced a significant increase in the number of cell nuclei expressing high levels of 5mC and 5hmC, but no change was observed in the number of cell nuclei expressing both marks in the TeO. The RN exhibited more cells expressing high levels of 5hmC in the nicotine-CPP group, while no changes were observed in the number of cell nuclei expressing both marks between treatments (E, F). Data obtained from four brains (n = 4 zebrafish per experimental condition) were analysed by two-way ANOVA. Vd/Vv: marker (F_2,18_: 3.526; p = 0.051), CPP (F_1,18_: 1.349; p = 0.2606) and marker × CPP interaction (F_2,18_: 1.14; p = 0.342); PTN: marker (F_2,18_: 6.715; p = 0.0066), CPP (F_1,18_: 0.1963; p = 0.663) and marker × CPP interaction (F_2,18_: 0.1131; p = 0.8937); Hb: marker (F_2,18_: 10.51; p = 0.0009), CPP (F_1,18_: 47.49; p < 0.0001) and marker × CPP interaction (F_2,18_: 0.09115; p = 0.9133); IPN: marker (F_2,18_: 5.136; p = 0.0172), CPP (F_1,18_: 1.124; p = 0.3031) and marker × CPP interaction (F_2,18_: 1.012; p = 0.3831); RN: marker (F_2,18_: 12.42; p = 0.0004), CPP (F_1,18_: 22.84; p = 0.0002) and marker × CPP interaction (F_2,18_: 2.396; p = 0.1196); TeO: marker (F_2,18_: 44.29; p < 0.0001), CPP (F_1,18_: 25.24; p < 0.0001) and marker × CPP interaction (F_2,18_: 3.709; p = 0.0448).

### 3.5 Immunofluorescence of 5hmC and NeuN in nuclei of the reward circuit of zebrafish

**FIGURE 8:**
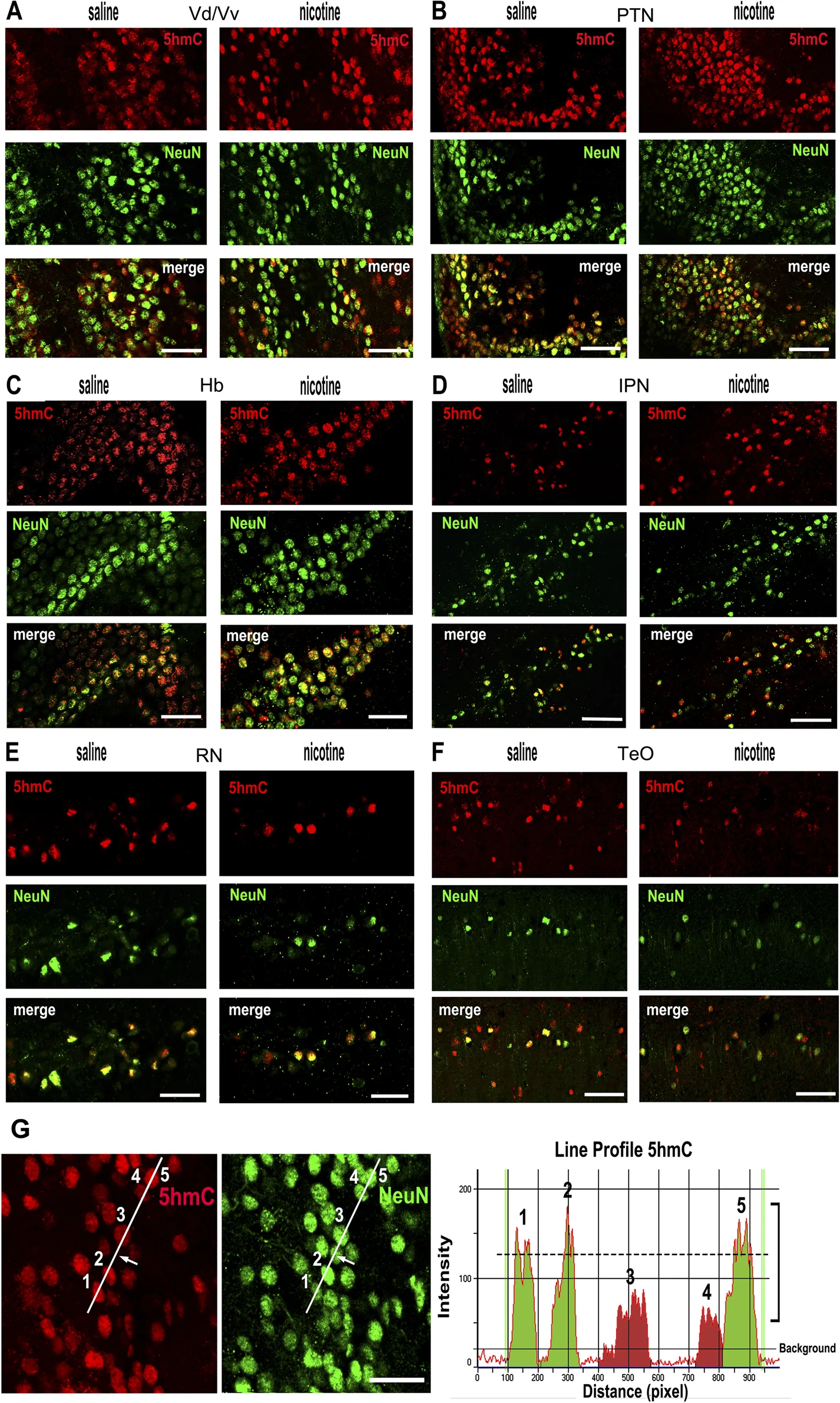
Fluorescent confocal images of cells labelled with 5hmC and NeuN in reward nuclei of zebrafish trained in the nicotine-CPP task. Images show cells expressing NeuN (green), 5hmC (red) and the double-positive nuclei for 5hmC and NeuN (yellow) in the dorsal and ventral nuclei of the ventral telencephalic area (Vd/Vv) **(A)**, posterior tuberal nucleus (PTN) **(B)**, habenula (Hb) **(C)**, interpeduncular nucleus (IPN) **(D)**, raphe nucleus (RN) **(E)** and tectum opticum (TeO) **(F)** (n = 4 zebrafish per experimental group). **(G)** Higher magnification images and profile of nuclei detected as positive (pink, above the 50% cut off value) and negative (green) by the counting algorithm. Cell nuclei were detected with hoechst (blue). Brackets indicate the range of cell nuclei positive for 5mC and 5hmC, from minimum to maximum specific staining; the dotted line indicates the cut off value. The arrow indicates a NeuN-positive nucleus, which is negative for 5hmC. Scale bars: 50 µm.

**FIGURE 9:**
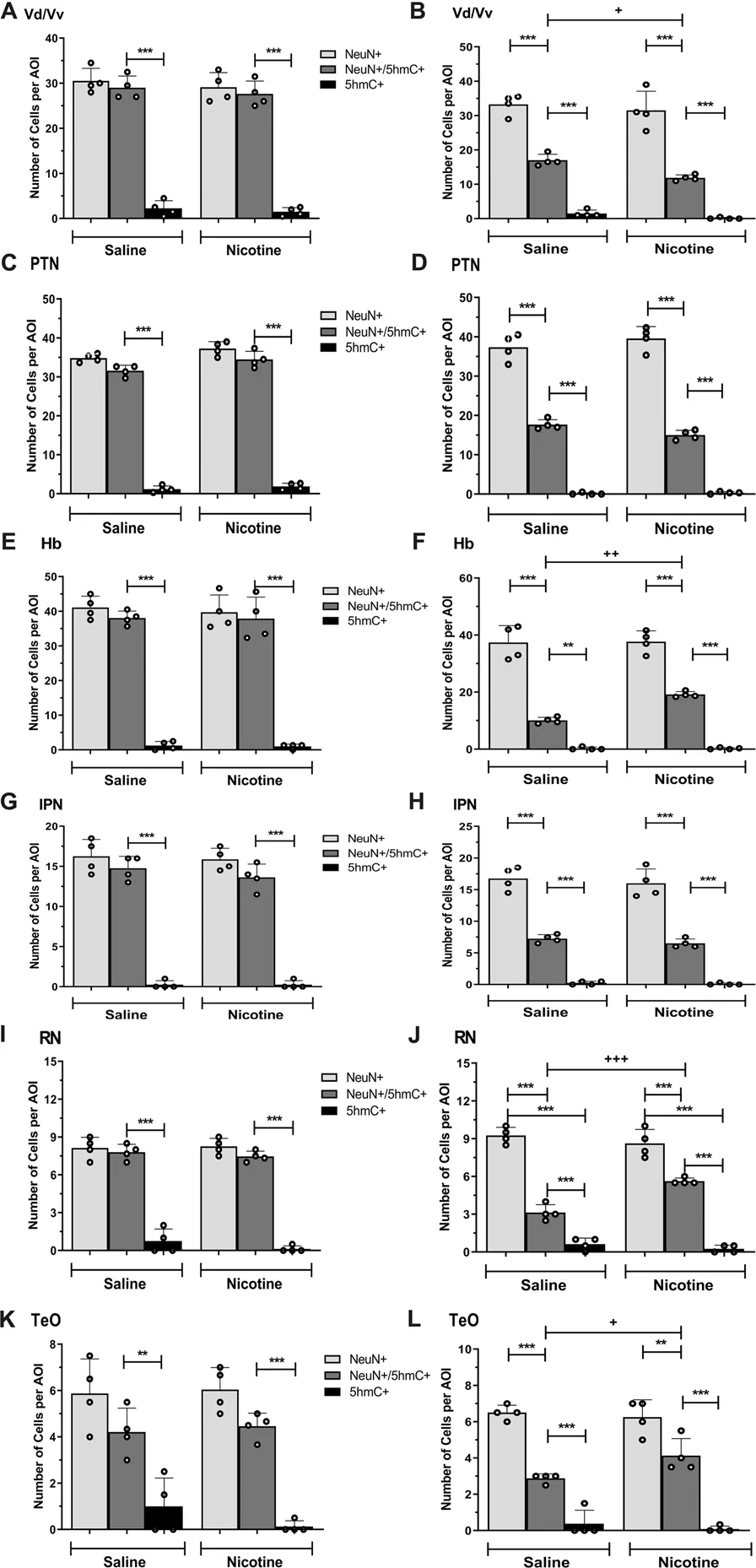
Quantification of cells labelled with 5hmC and NeuN in reward nuclei of zebrafish trained in the nicotine-CPP task. Graphs **(A,C,E,G,I, K)** show the total number of NeuN-positive nuclei (light gray bars), NeuN and 5hmC-double-positive nuclei (dark gray bars), and 5hmC-positive and NeuN-negative nuclei (black bars) per area of interest (AOI) in confocal images. Graphs **(B,D,F,G,L)** show the total number of NeuN-positive nuclei (light gray bars) and the number of nuclei that showed high 5hmC levels above a cutoff value (see stringent counting criteria in Methods) and were NeuN-positive (dark gray bars) or NeuN-negative (black bars) by AOI. **p < 0.01 and ***p < 0.001, comparisons intra-treatments; +p < 0.05, ++p < 0.01 and +++p < 0.001, comparisons inter-treatments (saline *vs* nicotine-CPP). Two-way ANOVA followed by Bonferroni *post hoc* test. **(A,B)** Vd/Vv: dorsal and ventral nuclei of the ventral telencephalic area; **(C,D)** PTN: posterior tuberal nucleus; **(E,F)** Hb: habenula; **(G,H)** IPN: interpeduncular nucleus; **(I,J)** RN: raphe nucleus; **(K,L)** TeO: tectum opticum (n = 4 zebrafish per experimental group).

To determine whether 5hmC was expressed in neurons, we assessed NeuN and 5hmC by immunofluorescence in reward circuit structures and the TeO of the brain of zebrafish trained in nicotine and saline-CPP tasks. Figure 8 shows representative confocal images of cell nuclei expressing 5hmC and NeuN, as well as cell nuclei doubly positive for both markers in the Vd/Vv, PTN, Hb, IPN, RN, and TeO. Most, though not all, of the cells expressing the neuronal marker NeuN also expressed varying levels of 5hmC along a gradient ranging from very low to high expression. The total number of NeuN- and 5hmC-positive cell nuclei were estimated and shown in Figures 9A,C,E,G,I,K. We observed that almost the same number of cell nuclei expressing NeuN were also positive for varying levels of 5hmC (which were clearly differentiated from background fluorescence). Our purpose was to evaluate whether nicotine-CPP modified the number of cells expressing high levels of 5hmC along with the NeuN marker. To that end, we counted 5hmC-positive nuclei, applying the stringent criteria described in Methods, that were also positive for NeuN (B, D, F, H, J, L). Half or even less than half of the doubly positive cell nuclei showed high levels of 5hmC. Significant increases in the number of double-positive cell nuclei (NeuN/high-5hmC) were observed in the Hb (F), the RN (J) and the TeO (L) of zebrafish trained in the nicotine-CPP task. The Vd/Vv (B) showed a significant decrease in the number of NeuN/high-5hmC-positive cell nuclei and the other reward nuclei did not showed significant changes between treatments (D, H). These results mirrored the data in Figure 5, which shows variable levels of 5hmC in virtually all cells and a higher number of cells with increased levels of this epigenetic mark, induced by nicotine CPP, in the same reward circuit areas. Most of the 5hmC-positive cells were neurons; between 1 and 2% of the 5hmC-positive nuclei were classified as non-neuronal cells. In the brain areas examined in this study, the number of NeuN-positive cell nuclei among zebrafish trained in the nicotine and saline-CPP tasks remained unchanged. We did not apply pixel-level intensity coefficients for double labelling determinations. Therefore, high-resolution microscopy will be needed to map the sub-nuclear distribution of 5hmC relative to 5 mC and NeuN-rich chromatin domains.

Data obtained from four brains (n = 4 zebrafish per experimental condition) were analysed by two-way ANOVA. Vd/Vv: marker (F_2,18_: 269.0; p < 0.0001), CPP (F_1,18_: 6.089; p = 0.0239) and marker × CPP interaction (F_2,18_: 1.145; p = 0.3404); PTN: marker (F_2,18_: 738.6; p < 0.0001), CPP (F_1,18_: 0.007; p = 0.9331) and marker × CPP interaction (F_2,18_: 3.058; p = 0.0719); Hb: marker (F_2,18_: 320.5; p < 0.0001), CPP (F_1,18_: 6.643; p = 0.019) and marker × CPP interaction (F_2,18_: 6.042; p = 0.0098); IPN: marker (F_2,18_: 331.3; p < 0.0001), CPP (F_1,18_: 1.156; p = 0.2965) and marker × CPP interaction (F_2,18_: 0.1416; p = 0.8689); RN: marker (F_2,18_: 359.4; p < 0.0001), CPP (F_1,18_: 3.724; p = 0.0695) and marker × CPP interaction (F_2,18_: 14.97; p = 0.0001); TeO: marker (F_2,18_: 172.5; p < 0.0001), CPP (F_1,18_: 0.7625; p = 0.394) and marker × CPP interaction (F_2,18_: 3.517; p = 0.0514).

## 4 Discussion

Epigenetic modifications such as DNA methylation and hydroxymethylation are mechanisms playing key roles in the regulation of gene expression and cellular function (Cheng et al., 2015). DNA 5 mC is a highly stable epigenetic modification. Maintaining DNA methylation patterns is critical, as 5 mC is the primary form of methylation found in adult neurons (Xie et al., 2012). The methylation status of DNA provides a fundamental epigenetic mechanism involved in the rewarding properties of several addictive drugs, including nicotine (Faillace and Bernabeu, 2022; Rustad et al., 2019). Our previous studies showed that nicotine-seeking behaviour is dependent on DNA demethylation principally in 5 mC (Pisera-Fuster et al., 2021). The first oxidation product of 5mC, 5hmC, has been reported to be effectively stable and significantly more abundant that other oxidation products of TET enzymes in the DNA demethylation pathway. Therefore, the evidence so far indicates that 5hmC may represent a distinct epigenetic mechanism for gene expression regulation rather than a transient intermediate for cytosine demethylation (Hahn et al., 2014; Guo et al., 2011). In this study, we focused our attention on 5hmC using a combination of behavioural and molecular analyses. Zebrafish trained in the nicotine-CPP exhibited typical behavioural parameters, including total distance swum and the number of entries into the nicotine-paired compartment (Pisera-Fuster et al., 2021; Kedikian et al., 2013). These parameters confirmed the robust CPP score induced by nicotine in zebrafish.

Nicotine reward induces upregulation of some subunits of the nAChRs in the zebrafish brain (Pisera-Fuster et al., 2019; Kedikian et al., 2013), a finding that reflects the high expression of nAChRs observed in the brains of individuals with tobacco use disorder (Govind et al., 2012). In this and previous studies, we observed that CHRNA6 and CHRNA7 mRNAs were upregulated by nicotine-CPP, suggesting a critical role for the α6 and α7 subunits of the nAChR in the establishment of nicotine reward. The *α4* subunit is frequently expressed together with the β2 subunit, forming the *α4β2* nAChR. All of these subunits are key neuronal subunits that control cognitive, locomotor, and anxiety-like behaviors in zebrafish. We have observed that levels of CHRNA4 and CHRNB2 mRNA were reduced and unchanged, respectively, in brain portions containing the reward circuit of zebrafish trained in the nicotine-CPP task (Pisera-Fuster et al., 2021; Kedikian et al., 2013). Thus, these and our previous results indicate that nicotine-rewarding effects are at least partially mediated by increases in the levels of CHRNA6 and CHRNA7 mRNA. Likewise, we observed that levels of GRIN1 mRNA were upregulated by nicotine-CPP. In a previous study, we have shown that *grin1* and *chrna7* were induced by demethylation of their gene promoters in the brain of zebrafish trained in the nicotine-CPP task (Pisera-Fuster et al., 2021).

The epigenetic genes we evaluated were selected based on their role in regulating 5mC and 5hmC levels. The short interval of 2 h after the end of the CPP test was selected to capture the rapid oxidation of methyl groups, as our primary goal was to characterize immediate epigenetic shifts induced by nicotine-CPP. Since the demethylation pathway eventually progresses toward other oxidation products—such as 5fC and 5caC—the early sampling window allowed us to isolate 5hmC, the critical first step in the cytosine demethylation pathway. Therefore, considering the expression of enzymes involved in the DNA methylation and demethylation cycle, our results clearly indicated that nicotine-CPP reduces the levels of DNMT mRNA in the zebrafish brain containing the reward nuclei and other brain structures. This suggests a reduction in the production of 5 mC. Previously, we have shown that nicotine-CPP induces transcriptional expression of DNMT1 while reducing transcriptional expression of DNMT3ab, which may reduce *de novo* DNA methylation (Pisera-Fuster et al., 2021). However, in this study we have observed a reduction in the expression level of DNMT1 and DNMT3ab mRNAs. This apparent contradiction could stem from differences in sampling kinetics. While the measurements in this study were performed 2 h after the CPP test, the previous study was performed 22 h later. Since DNMT1 primarily maintains the DNA methylation state during cell replication, its activity may not yet have been induced 2 h after the CPP test.

We have consistently observed that the levels of TET1 mRNA were upregulated, while the levels of TET2 mRNA were downregulated 2 h after the nicotine-CPP test. Given that TET2 has been implicated in the maintenance of global 5hmC in adult neural tissues (Joshi et al., 2022), it could be that the net effect induced by nicotine reward in the brain of zebrafish mainly involve increases in the levels of TET1 mRNA to promote the oxidation of 5mC to 5hmC in early intervals after the CPP-test. Furthermore, TET1 have been implicated in regulating 5hmC levels in learning and memory processes (Chen et al., 2017).

GADD45 was also included in this study because it helps recruit TETs to specific genomic sites and acts downstream of TET proteins to catalyse subsequent steps in the cytosine demethylation pathway. We observed a decrease in the level of GADD45 mRNA, which, together with reduced levels of TET2 mRNA, could suggest slower oxidation of 5hmC (Zipperly et al., 2021) in the brain of zebrafish trained in the nicotine-CPP task. In this study, the transcriptional expression of MeCP2 was also analysed because MeCP2 functions mainly as a repressor of gene expression by binding to 5 mC and other corepressor molecules; however, it can also function as an activator of different subsets of genes by interacting with 5hmCs and its role is context-dependent (Mellén et al., 2012). A decrease in the level of MeCP2 mRNA as well as a decrease in the 5 mC mark correlated with an increase in the levels of GRIN1 mRNA in the brain of zebrafish trained in the nicotine-CPP task*.* It has been described in mice that mutations of the *mecp2* gene involved in Rett syndrome, decrease the expression of the nAChR α-subunits (Leung et al., 2017). In our study, a decrease in the levels of MECP2 mRNA was associated with an increase in the levels of CHRNA6 and CHRNA7 mRNA, while levels of CHRNA4 mRNA were downregulated by nicotine-CPP. However, further investigation is needed to determine whether negative regulation of MeCP2 associated with nicotine CPP mediates nicotine reward-regulated gene expression. The Hb, a fundamental structure of the reward circuit of zebrafish, is involved in social conflict, fear, anxiety, reward, emotion and aversive responses to stimuli (Amo et al., 2014; Lammel et al., 2012). The Hb controls behaviour through the IPN and studies dealing with nicotine aversion and withdrawal has focused on the Hb-IPN pathway (Kim and Picciotto, 2023). In our study, the Hb showed an increase in the number of cell nuclei expressing high levels of 5mC and 5hmC in zebrafish trained in the nicotine-CPP task, suggesting important regulation of the DNA methylation and demethylation cycle and the activities of the DNMT and TET proteins. In the Hb, 5mC marks increased in some cell nuclei, while 5hmC marks increased in different cell nuclei. A third population of cell nuclei showed increased levels of both marks induced by nicotine-CPP. The percentage of cell nuclei that increased the 5hmC mark was higher than the percentage of cell nuclei that increased the 5 mC mark. These findings indicate the existence of subpopulations of neurons that responded differentially to nicotine-CPP. Cell nuclei in the PTN, homologous to the ventral tegmental area of the mammalian reward circuit containing dopaminergic neurons (Parker et al., 2013), and in the IPN, a primarily GABAergic nucleus, showed a similar pattern in the number of cell nuclei that contained high levels of 5mC and 5hmC. The number of cell nuclei containing high levels of 5hmC remained unchanged (or decreased slightly), while the number of cell nuclei containing high levels of the 5 mC mark was clearly reduced by nicotine-CPP. This may indicate a decrease in DNMT activity and an increase in TET activity that promotes the oxidation of both epigenetic marks. The medial Hb (mHb)-IPN pathway has a dense cholinergic innervation and exhibits a profuse and diverse expression of nicotinic receptors in mammalian vertebrates (Ciscato et al., 2025; Antolin-Fontes et al., 2015). In zebrafish, the Hb releases acetylcholine in the IPN, regulating its neuronal activity and AMPA glutamate receptors, through homomeric *α7*-nAChR, which are involved in changes in synaptic efficacy and neuronal plasticity during social behaviour (Kinoshita and Okamoto, 2023). Furthermore, the mHb has been implicated in nicotine withdrawal-induced anxiety behaviours via nAChR containing the *α6* subunit in mice (Pang et al., 2016). Thus, nicotine exposure during conditioning could have modified epigenetic methylation marks in habenular and IPN neurons. Nicotine-rewarding effects during conditioning could affect the DNA methylation status in the Hb and IPN differently in different neuronal populations. Nicotine has been shown to activate one population of neurons while inhibiting another population in the IPN; the distribution of these two populations of neurons does not appear to be segregated (Ciscato et al., 2025; Mondoloni et al., 2023). GABAergic neurons in the IPN project to the RN and the RN releases serotonin (as well as GABA and glutamate) modulating IPN activity and the dopaminergic nuclei in the reward circuit (Matsumata et al., 2025). The aforementioned reward nuclei form the Hb-RN and Hb-IPN-RN circuits, which are involved in amplified aversion, social conflict, and opioid and nicotine withdrawal in mice and zebrafish (Matsumata et al., 2025; Liang et al., 2024; Kinoshita and Okamoto, 2023; Pang et al., 2016; Amo et al., 2014). In our study, nicotine-CPP induced the recruitment of neurons with increased levels of the 5hmC mark in the RN in the midbrain. This suggests an increase in TET1 and a decrease in TET2, along with a decrease in GADD45, which is consistent with our findings on the effect of nicotine-CPP on the levels of these enzyme mRNAs. The Vd and Vv areas in the zebrafish subpallium, homologous to the extended amygdala, nucleus accumbens, and lateral septum, contain dopaminergic and GABAergic neurons, which are interconnected with the IPN and PTN (Armbruster et al., 2025). In zebrafish, the superior reticular nucleus (SRN) releases acetylcholine in the Hb and subpallium (Parker et al., 2013). Our findings indicated that the Vd/Vv areas showed a reduction in the number of cell nuclei containing high levels of the 5hmC mark. In line with our findings indicating increased levels of TET1 mRNA and unchanged 5mC levels, the decrease in 5hmC levels in cell nuclei of the Vd/Vv could be due to an increase in TET1 levels and the production of 5 fC and 5caC. The TeO is a major visuomotor hub that integrates polymodal sensory information in zebrafish (Baier and Scott, 2024). TeO, which is not considered a component of the reward circuit, showed an expression pattern of epigenetic DNA marks that suggested significant activation of the DNMT and TET1 proteins in different populations of a relatively low number of neurons recruited by nicotine-CPP in the parenchyma, outside the neurogenic PGZ area. Similar to the Hb, the TeO showed a higher percentage of cell nuclei that showed increases in the 5hmC mark than the percentage of cell nuclei that showed increases in the 5mC mark.

Our findings indicated that cell nuclei doubly positive for 5mC and 5hmC, at any level of staining intensity, observed in several brain areas related to nicotine reward, were more numerous than cell nuclei containing only 5mC or 5hmC. In most reward structures, the number of double-positive cell nuclei which showed increased levels of both epigenetic marks was unaffected by nicotine-CPP (with the exception of the Hb as described above). This suggests that the increase in DNA methylation and hydroxymethylation driven by nicotine CPP may occur in distinct neuronal populations within a particular area of the reward circuit (Andrade-Brito et al., 2024; Madrid et al., 2020). This pattern could be consistent with a scenario in which TET and DNMT are regulated independently across different neurons, rather than simultaneously within the same neurons (Tooley et al., 2023). Nevertheless, it cannot be ruled out that nicotine preference may modify the level and position of the 5mC and 5hmC marks within the same cell nuclei, causing dynamic changes in these cytosine marks in different regions of DNA, without changing the number of doubly marked cell nuclei. It has been described that 5hmC is especially enriched in neurons, constituting up to 30%–40% of the total cytosine modifications in neuronal DNA, compared to less than 10% in glia (Kozlenkov et al., 2013; Khare et al., 2012). Likewise, we found that nearly all cell nuclei positive for 5hmC were positive for NeuN. Zebrafish that exhibited nicotine reward-related behavioural changes showed more neuronal nuclei containing increased levels of 5hmC, particularly in the Hb, RN, and TeO (as reported in Figures 1, 8).

When DNA methylation and hydroxymethylation were estimated in the whole brain of zebrafish trained in the nicotine-CPP task, 5 mC levels decreased whereas 5hmC increased, suggesting net effects of nicotine reward on the zebrafish brain, which favours hydroxymethylation of DNA cytosines. By comparing this overall effect with the effect of nicotine-CPP on the nuclei of the reward circuit, where nicotine caused increases in both the 5mC and 5hmC marks, we can visualize that in the reward nuclei, the net effect of nicotine-CPP also favoured the demethylation of cytosines, either to 5hmC or to other oxidized intermediate cytosines. Some reward nuclei showed decreased levels of 5mC with no change in 5hmC, which could imply a reduction in cytosine methylation *via* reduced levels of DNMTs. Furthermore, additional effects of nicotine reward on other brain structures must be also considered, such as the effects of nicotine conditioning on epigenetic marks we observed in TeO neurons.

## 5 Concluding remarks

Epigenetic mechanisms play a critical role in the formation of drug-associated memories. In particular, DNA methylation and demethylation of cytosine residues modulate transcriptional programs underlying reward-related behaviours. Our results suggest that nicotine conditioned place preference in adult zebrafish is partly mediated by a decrease in the levels of DNMT1, DNMT3ab, TET2, GADD45, and MECP2 mRNA and an increase in the level of TET1 mRNA in an early interval after the nicotine-CPP. These effects are likely to reduce DNA methylation by decreasing cytosine methylation and by converting methylation to hydroxymethylation, thereby facilitating the establishment of nicotine-induced CPP. Furthermore, it is tempting to hypothesise that changes in cytosine methylation marks associated with nicotine-CPP could regulate genes related to nicotine reward, such as the genes encoding the α4, α6, and α7-nAChR subunits and NMDAR1 subunit, as we previously demonstrated for 5 mC. However, the cause-and-effect relationship between nicotine-CPP-induced changes on DNA methylation marks and gene expression needs further investigation. Following the acquisition and recovery of a memory and learning process, such as nicotine-associated reward, our findings indicated the recruitment of neurons that undergo significant epigenetic changes, such as hydroxymethylation of cytosine residues in some, but not all, interconnected nuclei of the reward circuit, especially in the Hb and RN. Nevertheless, nicotine-CPP effects on the cytosine methylation status appears to be very complex, since 5 mC marks were also increased in subpopulations of neurons in the Hb and TeO but decreased in the IPN and PTN by nicotine-CPP. These findings identify DNA methylation dynamics as important molecular changes associated to nicotine reward and suggest that 5hmC could be a potential target for interventions aimed at disrupting maladaptive drug-related memories.

## Data Availability

The raw data supporting the conclusions of this article will be made available by the authors, without on due reservation.
